# Longitudinal and cross-sectional predictors of sleep disturbance in a treatment follow-up sample with Tourette’s disorder

**DOI:** 10.3389/fpsyt.2025.1594347

**Published:** 2025-11-26

**Authors:** Maya S. Tooker, Kathryn E. Barber, Joseph F. McGuire, Flint M. Espil, Jordan T. Stiede, Jennifer S. Schild, Shannon M. Bennett, Matthew W. Specht, John T. Walkup, Douglas W. Woods, John Piacentini, Emily J. Ricketts

**Affiliations:** 1 Department of Psychiatry and Biobehavioral Sciences, University of California, Los Angeles, Los Angeles, CA, United States; 2 Department of Psychology, Marquette University, Milwaukee, WI, United States; 3 Department of Psychiatry and Behavioral Sciences, Johns Hopkins University School of Medicine, Baltimore, MD, United States; 4 Department of Psychiatry and Behavioral Sciences, Stanford University, Stanford, CA, United States; 5 Department of Psychology, Suffolk University, Boston, MA, United States; 6 Department of Psychiatry, Weill Cornell Medicine, New York, NY, United States; 7 Greenwich Anxiety, Greenwich, CT, United States; 8 Pritzker Department of Psychiatry and Behavioral Health, Ann and Robert H. Lurie Children's Hospital of Chicago, Chicago, IL, United States

**Keywords:** tics and Tourette syndrome, psychiatric comorbidity, impairment, sleep, medication

## Abstract

**Background:**

Sleep disturbance is common in individuals with Tourette’s disorder (TD). Tic symptoms, medication, functional impairment, and psychiatric comorbidity frequently contribute to sleep disturbance in children and adults with TD. However, long-term predictors of sleep disturbance in TD are not known. This study examined longitudinal and cross-sectional predictors of sleep disturbance in a treatment follow-up sample with TD.

**Methods:**

Eighty subjects who completed a 10-week randomized controlled trial of behavior therapy for tics in childhood (*M_age_* = 11.47, *SD* = 2.42 years) participate in follow-up evaluation on average, 11.17 (*SD* = 1.25) years after post-treatment assessment (*M_age_* = 22.87, *SD* = 2.70 years). At post-treatment (10-week) and long-term follow-up, an independent evaluator assessed tic severity and tic-related impairment using the Yale Global Tic Severity Scale. Parents provided demographic and medical history (e.g., tic medication and stimulant medication status) and rated ADHD severity. Children rated anxiety and depression. At follow-up, participants rated anxiety, depression, and ADHD severity, and reported tic and stimulant medication status. Multiple linear regression was performed to examine longitudinal and cross-sectional predictors of sleep disturbance (Pittsburgh Sleep Quality Index) at long-term follow-up.

**Results:**

tic-related impairment (*β* = .34, *p* = .014) at post-treatment positively predicted sleep disturbance at follow-up. Chronological age (*β* = .21, *p* = .041), anxiety severity (*β* = .40, *p* = .001), and ADHD severity (*β* = .31, *p* = .010) were positive cross-sectional predictors of sleep disturbance at follow-up.

**Conclusion:**

Results highlight the role of residual tic-related impairment following behavior therapy for tics delivered in childhood in addition to older age, anxiety severity, and ADHD severity in early adulthood in sleep disturbance in a treatment follow-up sample of adults with TD.

## 1 Introduction

Tourette’s disorder (TD) is a neurological condition marked by abrupt, repeated, involuntary movements (i.e., motor tics) and vocalizations (i.e., vocal tics) present for beyond one year (1). Tics typically first emerge between 4 and 8 years of age, with symptoms peaking in severity around 10 to 12 years, and thereafter waning during adolescence for most (2, 3). Though up to eighty percent of children experience a significant reduction in tic severity to mild levels or lower by early adulthood, twenty percent continue to experience clinically significant symptoms in adulthood - with females more likely to follow this non-remitting course (2, 4, 5). Thus, TD is more prevalent in males than females (ratio of 4:1) in childhood, though the sex ratio is less skewed toward male prevalence in adulthood (6).

TD exacts a significant toll on functioning and quality of life in children and adults, with psychiatric comorbidity exacerbating outcomes (5, 7). Psychiatric comorbidities are present in 85% of individuals with TD; attention-deficit/hyperactivity disorder (ADHD) is the most common co-occurring psychiatric condition, with obsessive-compulsive disorder (OCD), depression, and anxiety disorders also being prevalent (3). Comprehensive behavioral intervention for tics (CBIT) is a first-line intervention for TD (8), though just under half of children (48%) fail to exhibit positive treatment response and it showed limited benefit for co-occurring psychiatric symptoms and psychosocial functioning following acute treatment (9). Alpha-2 adrenergic agonists and antipsychotic medications are also efficacious and commonly prescribed (8), though a common side effect of alpha-2-agonists is sedation (10), and antipsychotic medications are associated with weight gain, sedation, metabolic syndrome, cognitive problems, and extrapyramidal symptoms, and to a lesser extent – insomnia (11, 12). Stimulant medications are frequently administered to address ADHD symptoms in patients with TD as well (13), but are commonly associated with negative side effects, such as reduced appetite, headache, stomach pain, insomnia, and sleep disorder (14, 15).

Sleep disturbance has received limited attention in TD. Sleep problems are prevalent at a rate of 34% in individuals with TD (16). Sleep disturbance per parent report composite (scored from 0 to 14) showed a significant age-related increase of 0.07 points per year among a large clinical cohort of individuals with TD between the ages of 5 and 26 years, suggesting sleep worsening with age (10, 17). Sleep disturbance serves as the linking mechanisms between tic severity and reduced tic-related quality of life (16). Sleep disturbance in individuals with TD is marked by insomnia, parasomnias (e.g., sleep walking, sleep talking), sleep-disordered breathing, and daytime sleepiness at respective prevalence rates of 32%, 27%, 15%, and 12% (16). Tics can also directly impact sleep – with 14% to 23% of individuals with TD reporting tic-related interference in falling asleep (18–21), and 14% to 32% reporting tic occurrence during sleep (22). Polysomnography, the gold standard for sleep measurement, confirms the presence of tics during sleep, though at reduced frequency, intensity, and complexity relative to waking periods (23–26). Common demographic and clinical correlates of sleep disturbance in children with TD include female sex, tic severity, impairment, and symptoms of co-occurring psychiatric conditions, most notably, ADHD, anxiety, and depression (27–32). In the few studies examining sleep in adult-only TD samples, older age, ADHD, overall impairment, and emotion dysregulation were associated with sleep disturbance (21, 33).

However, long-term predictors of sleep disturbance in TD are not known, and studies have not yet examined long-term and cross-sectional predictors of sleep disturbance in adulthood within the same sample. Such knowledge may yield differences in child and adult indicators of adult sleep outcomes, informing the potential utility of targeted interventions aimed at ameliorating sleep disturbance and improving quality of life in children and adults with TD. Therefore, this investigation examined candidate variables, including chronological age, sex, tic medication, stimulant medication, tic severity, tic-related impairment, anxiety severity, depression severity, and ADHD severity as both long-term and cross-sectional predictors of sleep disturbance in a treatment follow-up sample of adolescents and young adults with TD who had participated in a randomized controlled trial examining the efficacy of CBIT in childhood (9, 34). This sample was selected as there are limited longitudinal studies in clinical samples of adults with TD, and few are focused on long-term physical health outcomes (35). This sample allows for analysis of factors implicated in long-term sleep quality. As simpler statistical models offer greater interpretability and generalizability, this investigation used an exploratory variable selection approach for determining final predictors in order to achieve a model with reduced complexity and optimal goodness-of-fit (36).

## 2 Materials and methods

### 2.1 Participants

Participants were 80 individuals who completed a long-term follow-up evaluation (34) 11.17 years (*SD* = 1.25 years) after completion (i.e., 10-week post-treatment timepoint) of the original randomized controlled trial examining the efficacy of CBIT relative to Psychoeducation and supportive therapy [PST]) in 126 children and adolescents (9). See **Table 1** and Results for sample characteristics. Among the long-term follow-up participants, 38 were originally randomized to CBIT and 42 were randomized to PST. Among those who received CBIT, 55.6% (n = 21) were classified as treatment responders and 44.7% (n = 17) were non-responders, and among those who received PST, 14.3% (n = 6) were classified as treatment responders and 85.7% (n = 36) were non-treatment responders (See Procedure and Data Analysis section for definition of treatment responder and 34 for more details regarding sample characteristics).

**Table 1:** Demographic and clinical characteristics at baseline and post-treatment (10-week) for original (n = 126) and follow-up (n = 80) samples, and at long-term follow-up.

Characteristic	CBIT Baseline Characteristics							CBIT Post-treatment Characteristics								Long-term Follow-up Timepoint Characteristics (n = 80)	
	Long-term Follow-up Sample (n = 80)		Lost to Follow-up/Declined to Participate (n = 46)					Long-term Follow-up Sample (n = 80)			Lost to Follow-up/Declined to Participate (n = 46)						
	M	SD		M	SD	*t*	*p*	M	SD	M		SD	*t*	*p*		M	SD
Age	11.61	2.41		11.95	2.17	-0.80	.428	11.80	2.41	12.14		2.17	-0.80	.428		22.87	2.70
	n	%		n	%	*X* ^2^	*p*									n	%
Sex																	
Male	60	75.0		39	84.8%			–	–	–		–	–	–		60	75.0
Female	20	25.0		7	15.2%	1.13	.288	–	–	–		–	–	–		20	25.0
Tic Medication Status																	
On	29	36.3		17	40.0%			–	–	–		–	–	–		17	21.3
Off	51	63.7		29	63.0%	<0.001	>.999	–	–	–		–	–	–		54	67.5
Stimulant Medication Status																	
On	9	11.3		3	6.5%			–	–	–		–	–	–		12	15.0
Off	71	88.8		43	93.5%	0.31	.579	–	–	–		–	–	–		68	85.0
	M	SD		M	SD	*t*	*p*	M	SD	M		SD	*t*	*p*		M	SD
YGTSS Total Tic Severity	24.83	5.88		24.83	5.88	0.41	.686	19.20	8.18	18.94		7.54	0.16	.873		16.23	9.54
YGTSS Tic-related Impairment	23.65	8.25		23.65	8.82	-0.001	.999	13.72	10.13	14.69		10.54	-0.46	.644		10.00	10.77
SCARED/SCARED-Adult Total	18.73	11.00		17.39	10.82	0.66	.514	10.77	10.33	11.69		10.10	-0.44	.661		22.63	15.85
CDI/MFQ-Short Total	5.91	5.72		5.78	4.71	0.13	.893	4.09	4.98	3.83		4.45	0.27	.789		5.61	5.90
ADHD-RS-IV/ASRS Total	14.35	11.69		15.74	13.21	-0.61	.542	12.58	11.54	13.17		11.84	-0.25	.805		10.41	4.65
PSQI Total																7.24	2.46

CBIT, Comprehensive Behavioral Intervention for Tics; YGTSS, Yale Global Tic Severity Scale; SCARED, Screen for Child Anxiety Related Emotion Related Disorders; SCARED-Adult, An Adult Version of the Screen for Child Anxiety Related Disorders; CDI, Children’s Depression Inventory; MFQ, Mood and Feelings Questionnaire-Short; ADHD-RS-IV, ADHD-Rating Scale-IV; ASRS, Adult ADHD Self-Report Scale; PSQI, Pittsburgh Sleep Quality Index.

Inclusion criteria for the original trial included an age of 9 to 17 years; diagnosis of Tourette’s disorder, chronic motor tic disorder, or chronic vocal tic disorder, moderate or greater severity as measured by a Yale Global Tic Severity Scale (YGTSS; 37) Total score >13 (>9 for chronic motor tic disorder or chronic vocal tic disorder); fluency in English; and an intelligence quotient > 80. Exclusion criteria included the need for immediate treatment or change in current treatment for any of the co-occurring psychiatric disorders allowed at study entry: ADHD, obsessive–compulsive disorder, anxiety disorders, depressive disorders, or oppositional defiant disorder; changes in the dosage or schedule of any psychotropic medications within six weeks of study enrollment or planned changes or initiation during the study; unstable medical condition; current diagnosis of substance abuse/dependence; lifetime diagnosis of pervasive developmental disorder, mania or psychosis; or ≥ 4 sessions of habit reversal training (9). Of the original CBIT sample (n = 126), 30 were lost to follow and 16 declined to participate. See **Table 1** for baseline and post-treatment (10-week) demographic and clinical characteristics for participants who completed long-term follow-up (n = 80) and participants who were lost to follow up or declined to participate (n = 46). At follow-up, the sample ranged in age from 16 to 30 years (*M* = 22.87, *SD* = 2.70), was predominantly male (n = 60, 75.0%) and of non-Hispanic/Latino (n = 73, 91.2%) ethnicity. Participants endorsed white (n = 69, 86.3%), Black (n = 1, 1.3%), Asian (n = 4, 5.0%), multi-racial (n = 4, 5.0%), and other racial (n = 2, 2.5%) backgrounds. The majority of the sample were single/never married (n = 66, 82.5%) and had attained some college education or higher (n = 57, 71.3%). Half of the sample were employed (n = 40, 50.0%). See Espil et al. (34) for further methodological details.

### 2.2 Measures

*Pittsburgh Sleep Quality Index (PSQI).* The PSQI (61), administered at long-term follow-up is a 19-item self-report measure of sleep quality and disturbance over the previous month. Items are summed to produce seven subscale scores (sleep duration, sleep disturbance, sleep latency, daytime dysfunction due to sleepiness, sleep efficiency, overall sleep quality, and needs medication to sleep) are calculated from these items. These subscale scores are summed to yield a global sleep quality score. Higher scores indicate greater sleep disturbance. A PSQI global score greater than 5 is indicative of poor sleep quality (61). Forty-six participants in the long-term follow-up sample (57.5%) were above the cutoff for poor sleep quality. The PSQI has demonstrated acceptable reliability and validity (62–64). The internal consistency for PSQI Total in the present sample is.49.

See **Table 2** for detailed descriptions of measures evaluating predictor variables. including demographics (i.e., age, sex) and medical history (i.e., tic medication and stimulant medication status), tic severity and tic-related impairment, anxiety severity, depression severity, and ADHD symptom severity.

**Table 2:** Measures administered at baseline and long-term follow-up assessment timepoints.

Construct	Original CBIT trial measures	Long-term follow-up measures
Demographics and Medical History	Participants and/or parents provided demographic information and medical history, including tic medication and stimulation medication history.	Participants were asked about sociodemographics and medical history, including tic medication and stimulant medication use between the end of the CBIT trial and the long-term follow-up evaluation.
Tic Severity and Tic-related Impairment	*Yale Global Tic Severity Scale (YGTSS) – Total Tic Severity and Impairment.* The YGTSS (37) is a clinician-administered measure of tic severity and tic-related impairment in the previous week. Motor and vocal tics are assessed across five dimensions: number, frequency, intensity, complexity, and interference. Scores are summed to produce a Total Tic severity score ranging from 0 to 50. Tic-related impairment is rated on a single-dimension scale, also ranging from 0 to 50. Higher Total Tic and Impairment scores reflect greater severity and impairment, respectively. The YGTSS has strong reliability and validity, as evidenced by previous studies (37–39). The YGTSS Total Tic Severity score has an internal consistency of.92 in the present sample.	
Anxiety Severity	*Screen For Child Anxiety Related Emotional Disorders (SCARED).* The SCARED (40) is a parent- and child-report questionnaire measuring symptoms of anxiety disorders based on the DSM-IV. It contains 41 items rated on a scale of 0-2. Total scores range from 0-82, with higher scores indicating greater anxiety symptom severity and impairment. The SCARED has shown acceptable validity and reliability (40–43). The internal consistency of the SCARED total is.51 in this sample.	*An Adult Version of the Screen for Child Anxiety Related Disorders (SCARED-Adult).* The SCARED-Adult (44) is a 71-item adult self-report instrument assessing symptoms of anxiety disorders. Items are rated on a scale from 0-2, with higher scores indicating more severe anxiety. The SCARED-A demonstrates good internal consistency and convergent validity (44). The SCARED-A was completed at follow-up. We removed the 9 OCD items from this scale to prevent conceptual overlap among dependent variables. The internal consistency of the modified measure in the present sample was.94 with the OCD items included and.94 without.
Depression Severity	*Children’s Depression Inventory (CDI).* The CDI (45, 46) is a 27-item self-report measure that assesses emotional, cognitive, behavioral, and somatic symptoms that characterize depression. Higher scores indicate more severe depression. The CDI has strong psychometric properties (47–49). The internal consistency of the CDI total score in this sample is.86 in the present sample.	*Mood and Feelings Questionnaire – Short Version (MFQ).* The MFQ (50, 51) is a 13-item measure of depression over the prior two weeks. Each item is rated on a 3-point scale with higher total scores indicating greater depressive symptoms. The MFQ demonstrates high internal consistency and strong validity in adolescent and emerging adulthood samples (51, 52). The internal consistency of the MFQ Total in this sample was.93.
ADHD severity	*ADHD Rating Scale-IV (ADHD-RS-IV).* The ADHD-RS-IV (53, 54) is an 18-item parent-report measure assessing child ADHD symptoms and corresponds with DSM-IV diagnostic criteria. Each item is scored on a scale from 0-3, with higher scores indicating higher ADHD symptom severity. The ADHD RS-IV has good reliability and validity (55, 56). The internal consistency of the ADHD-RS-IV total score is.95 in this sample.	*Adult ADHD Self-Report Scale (ASRS).* The ASRS (57) is a six-item screening measure used to assess ADHD symptoms over the previous six months. Items are consistent with DSM-IV diagnostic criteria. Previous studies support the validity (58, 59), internal consistency (58), and test-retest reliability (60) of the ASRS. The internal consistency of the ASRS in the current sample was.77.

### 2.3 Procedure

Original CBIT Trial: Following institutional review board parent permission, child assent, and screening assessment procedures, participants and their parents completed a baseline evaluation for a 10-week randomized, controlled clinical trial evaluating the efficacy of CBIT relative to PST (9). CBIT is an 8-session blended intervention delivered over 10 weeks, involving habit reversal training, function-based assessment and intervention, relaxation training, and behavioral rewards (65). PST is a structured intervention providing supportive therapy and psychoeducation about tics developed to control attention and time (9). Treatment was delivered at three sites (University of California, Los Angeles [n = 45], University of Wisconsin-Milwaukee/Marquette University [n = 40] and Johns Hopkins University [n = 41]). This was followed a post-treatment evaluation during which a trained independent evaluator (IE) assessed tic severity and tic-related impairment (Yale Global Tic Severity Scale; YGTSS; 37) and rated degree of participant improvement in global illness-related functioning using the Clinical Global Impressions-Improvement Scale [CGI-I]; 66). Parents provided demographics and medical history and rated child behavior, including ADHD symptom severity (ADHD Rating Scale-IV; ADHD-RS-IV; 53, 54). Children rated anxiety severity (Screen for Child Anxiety Related Emotional Disorders; SCARED; 40) and depressive symptom severity (Children’s Depression Inventory; CDI; 45, 46).

Long-term Follow-up Assessment: At long-term follow-up assessment, participants from the original trial were recruited from two of the three original CBIT sites: University of California, Los Angeles (n = 32), Marquette University (n = 22), and Weill Cornell University (n = 26) due to the move of the original Johns Hopkins University principal investigator to that university. Adults provided institutional review board-approved informed consent for their participation. The three child participants (aged 16–17 years) provided assent, and their parents provided parent permission. The follow-up evaluation was completed in person or via web-based videoconferencing as needed. Independent evaluators (IEs) with a bachelor’s degree or higher, masked to original treatment assignment, and trained to reliability on clinical interviews (i.e., received didactic training and achieved reliable ratings on two consecutive interview administrations; see 34), evaluated psychiatric diagnosis (Mini International Neuropsychiatric Interview; MINI; 67), tic severity, and tic-related impairment (YGTSS). Participants rated sleep (PSQI Total), anxiety severity (An Adult Version of the Screen for Child Anxiety Related Disorders; SCARED-Adult; 44), depressive symptom severity (Mood and Feelings Questionnaire – Short Version; MFQ; 50), and ADHD symptom severity (Adult ADHD Self-Report Scale; ASRS; 57).

### 2.4 Data analysis

Analyses were conducted using SPSS 28.0. Descriptive statistics, including means and frequencies, are presented to characterize the sample with respect to demographics. Multiple linear regression was performed to examine whether post-treatment (i.e., 10-week timepoint of original CBIT trial) variables (including sex, tic medication status, stimulant medication status, YGTSS Tic Severity Total, YGTSS Impairment, SCARED Total, CDI Total, and ADHD-RS-IV Total) were predictors of the PSQI Total score at long-term follow-up. Multiple linear regression was also performed to examine whether variables measured at long-term follow-up (sex, tic medication status since the trial ended, stimulant medication status since the trial ended, YGTSS Tic Severity Total, YGTSS Tic-related Impairment Total, SCARED-Adult Total, CDI Total, and ADHD-RS-IV Total) were significant predictors of PSQI Total score. In both analyses, treatment assignment (CBIT versus PST), and treatment responder status (i.e., CGI-I rating of ‘much improved’ or ‘very much improved’ relative to a rating of ‘improved,’ ‘minimally improved,’ ‘no change,’ ‘minimally worse,’ ‘much worse,’ or ‘very much worse’) were included as covariates to account for prior tic treatment effects.

Then these analyses were repeated using multiple linear regression with backward elimination. Covariates (treatment assignment, treatment responder status) were entered into the model at each step of the backward elimination process, while predictor variables with a p-value of <.05 were removed from the model at each step in ordered fashion. Model fit was assessed at each step until achieving the final reduced model. Final reduced models were selected based on parsimony (i.e., simplest model that maintains goodness-of-fit) (36). We report adjusted R^2^, indicating the proportion of variance explained by the model adjusted for the number of predictors in the model. A higher R^2^ value indicates greater goodness-of-fit based on the predictors in the model (68). The variance-inflation factor (VIF) is reported as a measure of multicollinearity. Lower VIF scores indicate reduced collinearity among predictor variables. VIF scores above 10 indicate substantial multicollinearity among independent variables (69).

## 3 Results

### 3.1 Comparison of demographic and clinical characteristics in long-term follow-up sample and those lost-to follow-up or who declined to participate

There were no significant differences in demographic and clinical characteristics (i.e., age, sex, tic medication status, stimulant medication status, YGTSS Total Tic Severity and Tic-related Impairment scores, SCARED Total, CDI Total, ADHD-RS-IV Total) at baseline of the original CBIT trial between participants who completed long-term follow-up and participants who were lost to follow-up or declined to participate (*p* = >.999 –.288). There were no significant differences in clinical chacteristics at post-treatment (10-week) timepoint of the original CBIT trail between participants who completed long-term follow-up and participants who were lost to follow up or declined to participate (*p* = .644 –.873). For bivariate correlations among clinical measures at post-treatment (10-week) and long-term follow-up see **Supplementary Tables 1**, **2**.

### 3.2 Post-treatment predictors of sleep disturbance at long-term follow up

In the full model, there were no significant post-treatment (10-week) predictors of PSQI Total at long-term follow up (see **Table 3** and **Supplementary Table 3**). The full model explained 16% of the variance in PSQI Total score at long-term follow-up. In the final model, three predictors were retained, including sex (i.e., female), YGTSS Tic-related Impairment score, and ADHD-RS-IV Total score. Of these, YGTSS Tic-related Impairment score (*β* = .34) was a statistically significant predictor of PSQI Total score (see **Table 3** and **Supplementary Table 3**). Sex and ADHD-RS-IV Total score were retained in the final model despite p-values >.05 based on parsimony; together, these three variables reflected the simplest model while preserving adjusted R^2^. The final model explained 15% of the variance in PSQI Total score.

**Table 3:** Post-CBIT (10-week) predictors of PSQI total at long-term follow-up.

Predictors	Full model			Final model		
	*β*	*p*	VIF	*β*	*p*	VIF
Constant		.012			.004	
Treatment Assignment	.06	.646	1.41	.05	.697	1.34
Treatment Responder Status	-.08	.607	2.15	.02	.883	1.67
Age at post-treatment	-.01	.960	1.32			
Sex	-.18	.149	1.22	-.18	.130	1.07
Tic Medication	.15	.236	1.22			
Stimulant Medication	-.21	.075	1.11			
YGTSS Total Tic Severity Score	-.19	.253	2.29			
YGTSS Tic-related Impairment Score	.28	.094	2.16	.34	.014	1.43
SCARED Total	.06	.652	1.32			
CDI Total	.12	.436	2.00			
ADHD-RS-IV Total	.20	.151	1.59	.17	.149	1.13
Adjusted R²	.16			.15		

Full model refers to multiple linear regression including covariates (treatment assignment and treatment responder status) entered together with predictor variables. Final model refers to multiple linear regression with backward elimination – with covariates (treatment assignment, treatment responder status) entered into the model at each step and predictor variables with a p-value of <.05 removed from the model at each step in ordered fashion.

Treatment Assignment refers to randomization to Comprehensive Behavioral Intervention for Tics (coded as 1) versus Psychoeducation and Supportive Therapy (coded as 0). Treatment Responder Status refers to Clinical Global Impressions CGI-I rating of ‘much improved’ or ‘very much improved’ (coded as 1) relative to a rating of ‘improved,’ ‘minimally improved,’ ‘no change,’ ‘minimally worse,’ ‘much worse,’ or ‘very much worse’ (coded as 0). Sex is coded as 1 (male) and 0 (female). Medications are coded as 1 (on medication) and 0 (off medication).

YGTSS, Yale Global Tic Severity Scale; SCARED, Screen for Child Anxiety Related Emotional Disorders; CDI, Children’s Depression Inventory; ADHD-RS-IV, ADHD-Rating Scale-IV; PSQI, Pittsburgh Sleep Quality Index.

*β =* standardized beta (regression) coefficient, which ranges from -1 to +1; values nearer -1 or 1 indicate a stronger association between predictor and dependent variable. The alpha level was set to.05. VIF = variance inflation factor; lower scores indicate reduced multicollinearity among predictor variables. Adjusted R^2^ = predictive power of a model adjusted for the number of predictors in the model; higher values indicate greater predictive power based on the predictors in the model.

### 3.3 Cross-sectional predictors of sleep disturbance at long-term follow-up

In the full model, SCARED-Adult Total score (*β* = .38) was a significant cross-sectional predictor of PSQI Total at long-term follow-up (see **Table 4** and **Supplementary Table 4**). The full model explained 44% of the variance in PSQI Total score at long-term follow-up. Three predictors were retained in the final reduced model; chronological age, SCARED-Adult Total score (*β* = .40), ASRS Total score (*β* = .31)) were positive predictors of PSQI Total score at long-term follow-up (See **Table 4** and **Supplementary Table 4**). The final reduced model explained 42% of the variance in PSQI Total score.

**Table 4:** Cross-sectional predictors of PSQI total at long-term follow-up.

Predictors	Full model			Final model		
	*β*	*p*	VIF	*β*	*p*	VIF
Constant		.855			661	
Treatment Assignment	-.05	.676	1.39	-.09	.442	1.25
Treatment Responder Status	-.28	.025	1.55	-.34	.004	1.29
Age	.22	.041	1.11	.21	.041	1.05
Sex	-.09	.406	1.16			
Tic Medication	.17	.118	1.19			
Stimulant Medication	-.05	.611	1.15			
YGTSS Total Tic Severity Score	-.24	.108	2.26			
YGTSS Tic-related Impairment Score	.27	.079	2.35			
SCARED-Adult Total	.38	.006	1.83	.40	.001	1.36
MFQ-Short Total	.07	.677	2.69			
ASRS Total	.21	.143	2.14	.31	.010	1.37
Adjusted R²	.44			.42		

Full model refers to multiple linear regression including covariates (treatment assignment and treatment responder status) entered together with predictor variables. Final model refers to multiple linear regression with backward elimination – with covariates (treatment assignment, treatment responder status) entered into the model at each step and predictor variables with a p-value of <.05 removed from the model at each step in ordered fashion.

Treatment Assignment refers to randomization to Comprehensive Behavioral Intervention for Tics (coded as 1) versus Psychoeducation and Supportive Therapy (coded as 0). Treatment Responder Status refers to Clinical Global Impressions CGI-I rating of ‘much improved’ or ‘very much improved’ (coded as 1) relative to a rating of ‘improved,’ ‘minimally improved,’ ‘no change,’ ‘minimally worse,’ ‘much worse,’ or ‘very much worse’ (coded as 0). Sex is coded as 1 (male) and 0 (female). Medications are coded as 1 (on medication) and 0 (off medication).

YGTSS, Yale Global Tic Severity Scale; SCARED-Adult, An Adult Version of the Screen for Child Anxiety Related Disorders; MFQ, Mood and Feelings Questionnaire-Short; ASRS, Adult ADHD Self-Report Scale; PSQI, Pittsburgh Sleep Quality Index.

*β =* standardized beta (regression) coefficient, which ranges from -1 to 1; values nearer -1 or 1 indicate a stronger association between predictor and dependent variable. The alpha level was set to.05. VIF = variance inflation factor; lower scores indicate reduced multicollinearity among predictor variables. Adjusted R^2^ = predictive power of a model adjusted for the number of predictors in the model; higher values indicate greater predictive power based on the predictors in the model.

## 4 Discussion

The present study examined both long-term and cross-sectional predictors of sleep disturbance in early adulthood in a treatment-follow-up sample with TD. Findings showed residual tic-related impairment following acute behavior therapy for tics was a significant predictor of sleep disturbance in early adulthood. Older chronological age, in addition to greater anxiety severity and ADHD severity were significant cross-sectional predictors of sleep disturbance in early adulthood.

The association between residual tic-related impairment and sleep disturbance in early adulthood aligns with cross-sectional findings showing greater parent-rated tic-related impairment in children with TD and comorbid sleep disorders relative to TD alone (30) and correlations between reduced tic-related quality of life and greater sleep disturbance (16). In the Li et al. (16) study, greater sleep disturbance was associated with reduced quality of life broadly and across several domains, including cognitive, obsessive-compulsive, physical/activities of daily living, and psychological. As the YGTSS tic-related impairment item used in the present study is global it is unclear which aspects of tic-related impairment may be more strongly associated with sleep disturbance. This finding may suggest the need for more direct intervention targeting tic-related impairment. However, it is important to note that although CBIT failed to show significant positive effects on secondary psychiatric symptoms and psychosocial functioning relative to PST in the acute treatment period, for positive treatment responders, there were positive improvements in these outcomes at 3- and 6-month follow-up.

The cross-sectional association between older chronological age and sleep disturbance in early adulthood aligns with general age-related trends in sleep patterns in the general population and TD samples, which indicate sleep worsens with older age (17, 70, 71; Ricketts et al., 2022). The finding that ADHD symptom severity was a significant predictor of sleep disturbance is consistent with the wealth of studies showing links between ADHD and sleep disturbance in adults, including difficulties falling asleep, nighttime awakenings, reduced sleep quality, and daytime sleepiness (72, 73). Shared neural correlates (i.e., reduced gray matter volumes in the middle frontal and inferior frontal gyri, amygdala, striatum, and insula) are one potential mechanism underlying the association between ADHD and sleep disruption (74). The association found between anxiety and sleep disturbance at follow-up is not surprising due to well-established links between anxiety and sleep disturbance broadly (75) and in individuals with TD (27). Anxiety may increase emotional and physiological arousal impeding sleep and potentially exacerbating tics (76, 77; 78). One potential mechanism linking anxiety and sleep disturbance is the hypothalamic-pituitary-adrenal (HPA) axis, which modulates the stress response system (79). There is also preliminary support for the role of the HPA axis in TD (80).

The present study has notable limitations. The PSQI had low internal consistency in this sample, which could impact the validity of our results. We lack objective measurement of sleep disturbance. Also, the modest sample size might limit the generalizability of these findings to the broader population of individuals with TD. Additionally, as this study includes a follow-up sample stemming from a treatment seeking sample, results may not generalize to the broader population of individuals with TD. Further, this analysis does not address bidirectionality in associations between predictors and sleep disturbance. Moreover, this analysis does not examine change in sleep disturbance across acute behavior therapy for tics (CBIT), limiting conclusions which may be drawn regarding the treatment implications of this research.

In sum, the present study highlights the role of residual tic-related impairment following acute behavior therapy for tics and chronological age, anxiety, and ADHD symptoms on sleep disturbance in early adulthood. Future research should seek to understand directional associations and mechanistic links between TD, demographic and clinical chacteristics, and sleep disturbance. Future prospective trials are also needed to examine the degree to which CBIT improves sleep in a sample selected based on presence of comorbid TD and clinical threshold sleep disturbance using validated subjective and objective sleep measures.

## Data Availability

The raw data supporting the conclusions of this article will be made available by the authors, without undue reservation.
