# Supplementary material for: Longitudinal and cross-sectional predictors of sleep disturbance in a treatment follow-up sample with Tourette’s disorder
Source: Front Psychiatry. 2025 Nov 26;16:1594347. doi: 10.3389/fpsyt.2025.1594347 (PMC12690208; doi:10.3389/fpsyt.2025.1594347)
Supplement: Supplementary file 1 [file Table1.docx]

Supplementary Table 1

*Pearson Bivariate Correlations among Post-treatment (10-week) Predictor Measures and PSQI Total at Long-Term Follow-up*

| Variable | 1 | 2 | 3 | 4 | 5 | 6 | 7 | 8 | 9 | 10 |
| --- | --- | --- | --- | --- | --- | --- | --- | --- | --- | --- |
| 1. Age at Post-treatment (10-week) | __ |  |  |  |  |  |  |  |  |  |
| 2. Sex | -.16 | __ |  |  |  |  |  |  |  |  |
| 3. Tic Medication | .28* | .14 | __ |  |  |  |  |  |  |  |
| 4. Stimulant Medication | -.02 | -.07 | -.02 | __ |  |  |  |  |  |  |
| 5. YGTSS Total Tic Severity Score | .16 | .03 | .17 | -.04 | __ |  |  |  |  |  |
| 6. YGTSS Tic-related Impairment Score | .19 | -.16 | .23* | -.03 | .60** | __ |  |  |  |  |
| 7. SCARED Total | .26* | -.05 | .20 | .02 | .29* | .21 | __ |  |  |  |
| 8. CDI Total | .20 | -.15 | .25* | .14 | .33** | .51** | .34** | __ |  |  |
| 9. ADHD-RS-IV Total | -.15 | .04 | .06 | .19 | .24* | .21 | -.02 | .45** | __ |  |
| 10. PSQI Total | .12 | -.22 | .22 | -.14 | .18 | .40** | .16 | .36** | .22 | __ |

*Note.* **p* < .05; ** *p* < .001; Sex is coded as 1 (male) and 0 (female). Medications are coded as 1 (on medication) and 0 (Not on medication).

YGTSS = Yale Global Tic Severity Scale; SCARED = Screen for Child Anxiety Related Emotional Disorders; CDI = Children’s Depression Inventory; ADHD-RS-IV = ADHD-Rating Scale-IV; PSQI = Pittsburgh Sleep Quality Index.

Supplementary Table 2

*Pearson Bivariate Correlations among Cross-sectional Predictor Measures and PSQI Total at Long-Term Follow-up*

| Variable | 1 | 2 | 3 | 4 | 5 | 6 | 7 | 8 | 9 | 10 |
| --- | --- | --- | --- | --- | --- | --- | --- | --- | --- | --- |
| 1. Age | __ |  |  |  |  |  |  |  |  |  |
| 2. Sex | -.17 | __ |  |  |  |  |  |  |  |  |
| 3. Tic Medication | .28* | .14 | __ |  |  |  |  |  |  |  |
| 4. Stimulant Medication | -.08 | -.07 | -.02 | __ |  |  |  |  |  |  |
| 5. YGTSS Total Tic Severity Score | .07 | -.03 | .01 | -.18 | __ |  |  |  |  |  |
| 6. YGTSS Tic-related Impairment Score | .01 | -.12 | .02 | -.20 | .72** | __ |  |  |  |  |
| 7. SCARED-Adult Total | .02 | -.05 | -.03 | -.33* | .13 | .20 | __ |  |  |  |
| 8. MFQ-Short Total | .02 | -.13 | .14 | -.17 | .09 | .27* | .64** | __ |  |  |
| 9. ASRS Total | .06 | -.04 | .06 | -.12 | .15 | .26* | .50** | .67** | __ |  |
| 10. PSQI Total | .28* | -.22 | .22 | -.14 | .15 | .33** | .51** | .49** | .47** | __ |

*Note.* **p* < .05; ** *p* < .001; Sex is coded as 1 (male) and 0 (female). Medications are coded as 1 (on medication) and 0 (Not on medication).

YGTSS = Yale Global Tic Severity Scale; SCARED-Adult = An Adult Version of the Screen for Child Anxiety Related Disorders; MFQ = Mood and Feelings Questionnaire-Short; ASRS = Adult ADHD Self-Report Scale; PSQI = Pittsburgh Sleep Quality Index.

Supplementary Table 3

*Post-CBIT (10-week) Predictors of PSQI Total at Long-term Follow-Up without and with Backward Elimination*

| Model | Predictors | B | SE | *β* | *t* | *p* | VIF | Adj. R² |
| --- | --- | --- | --- | --- | --- | --- | --- | --- |
| 1 | Full Model |  |  |  |  |  |  | .16 |
|  | Constant | 6.09 | 2.36 |  | 2.58 | .012 |  |  |
|  | Treatment Assignment | 0.36 | 0.79 | .06 | 0.46 | .646 | 1.41 |  |
|  | Treatment Responder Status | -0.53 | 1.02 | -.08 | -0.52 | .607 | 2.15 |  |
|  | Age | -0.01 | 0.16 | -.01 | -0.05 | .960 | 1.32 |  |
|  | Sex | -1.23 | 0.84 | -.18 | -1.46 | .149 | 1.22 |  |
|  | Tic Medication | 0.91 | 0.76 | .15 | 1.20 | .236 | 1.22 |  |
|  | Stimulant Medication | -1.99 | 1.09 | -.21 | -1.81 | .075 | 1.11 |  |
|  | YGTSS Total Tic Severity Score | -0.07 | 0.06 | -.19 | -1.16 | .253 | 2.29 |  |
|  | YGTSS Tic-related Impairment | 0.08 | 0.05 | .28 | 1.70 | .094 | 2.16 |  |
|  | SCARED Total | 0.02 | 0.04 | .06 | 0.45 | .652 | 1.32 |  |
|  | CDI Total | 0.07 | 0.09 | .12 | 0.78 | .436 | 2.00 |  |
|  | ADHD-RS-IV Total | 0.05 | 0.04 | .20 | 1.46 | .151 | 1.59 |  |
|  |  |  |  |  |  |  |  |  |
| 2 | Final Model |  |  |  |  |  |  | .15 |
|  | Constant | 4.93 | 1.67 |  | 2.95 | .004 |  |  |
|  | Treatment Assignment | 0.30 | 0.77 | .05 | 0.39 | .697 | 1.34 |  |
|  | Treatment Responder Status | 0.13 | 0.91 | .02 | 0.15 | .883 | 1.67 |  |
|  | Sex | -1.22 | 0.79 | -.18 | -1.53 | .130 | 1.07 |  |
|  | YGTSS Tic-related Impairment Score | 0.10 | 0.04 | .34 | 2.52 | .014 | 1.43 |  |
|  | ADHD-RS-IV Total | 0.05 | 0.03 | .17 | 1.46 | .149 | 1.13 |  |

*Note.* Full model refers to multiple linear regression including covariates (treatment assignment and treatment responder status) entered together with predictor variables. Final model refers to multiple linear regression with backward elimination – with covariates (treatment assignment, treatment responder status) entered into the model at each step and predictor variables with a p-value of < .05 removed from the model at each step in ordered fashion.

Treatment Assignment refers to randomization to Comprehensive Behavioral Intervention for Tics (coded as 1) versus Psychoeducation and Supportive Therapy (coded as 0). Treatment Responder Status refers to Clinical Global Impressions CGI-I rating of ‘much improved’ or ‘very much improved’ (coded as 1) relative to a rating of ‘improved,’ ‘minimally improved,’ ‘no change,’ ‘minimally worse,’ ‘much worse,’ or ‘very much worse’ (coded as 0). Sex is coded as 1 (male) and 0 (female). Medications are coded as 1 (on medication) and 0 (off medication).

YGTSS = Yale Global Tic Severity Scale; SCARED = Screen for Child Anxiety Related Emotional Disorders; CDI = Children’s Depression Inventory; ADHD-RS-IV = ADHD-Rating Scale-IV; PSQI = Pittsburgh Sleep Quality Index.

*β =* standardized beta (regression) coefficient, which ranges from -1 to +1; values nearer -1 or 1 indicate a stronger association between predictor and dependent variable. The alpha level was set to .05. VIF = variance inflation factor; lower scores indicate reduced multicollinearity among predictor variables. Adjusted R^2^ = predictive power of a model adjusted for the number of predictors in the model; higher values indicate greater predictive power based on the predictors in the model.

Supplementary Table 4

*Cross-sectional Indicators of PSQI Total at Long-Term Follow-Up without and with Backward Elimination*

| Model | Predictors | B | SE | *β* | *t* |  | *p* | VIF | Adj. R² |
| --- | --- | --- | --- | --- | --- | --- | --- | --- | --- |
| 1 | Full Model |  |  |  |  |  |  |  | .44 |
|  | Constant | -0.54 | 2.93 |  | -0.18 |  | .855 |  |  |
|  | Treatment Assignment | -0.29 | 0.69 | -.05 | -0.42 |  | .676 | 1.39 |  |
|  | Treatment Responder Status | -1.77 | 0.77 | -.28 | -2.31 |  | .025 | 1.55 |  |
|  | Age | 0.24 | 0.12 | .22 | 2.10 |  | .041 | 1.11 |  |
|  | Sex | -0.61 | 0.73 | -.09 | -0.84 |  | .406 | 1.16 |  |
|  | Tic Medication | 1.19 | 0.75 | .17 | 1.59 |  | .118 | 1.19 |  |
|  | Stimulant Medication | -0.45 | 0.88 | -.05 | -0.51 |  | .611 | 1.15 |  |
|  | YGTSS Total Tic Severity Score | -0.08 | 0.05 | -.24 | -1.64 |  | .108 | 2.26 |  |
|  | YGTSS Tic-related Impairment Score | 0.08 | 0.04 | .27 | 1.80 |  | .079 | 2.35 |  |
|  | SCARED-Adult Total | 0.07 | 0.03 | .38 | 2.85 |  | .006 | 1.83 |  |
|  | MFQ-Short Total | 0.03 | 0.08 | .07 | 0.42 |  | .677 | 2.69 |  |
|  | ASRS Total | 0.14 | 0.09 | .21 | 1.49 |  | .143 | 2.14 |  |
|  |  |  |  |  |  |  |  |  |  |
| 2 | Final Model |  |  |  |  |  |  |  | .42 |
|  | Constant | -1.23 | 2.79 |  | -0.44 |  | .661 |  |  |
|  | Treatment Assignment | -0.51 | 0.66 | -0.09 | -0.78 |  | .442 | 1.25 |  |
|  | Treatment Responder Status | -2.12 | 0.71 | -0.34 | -3.00 |  | .004 | 1.29 |  |
|  | Age | 0.24 | 0.11 | 0.21 | 2.09 |  | .041 | 1.05 |  |
|  | SCARED-Adult Total | 0.08 | 0.02 | 0.40 | 3.42 |  | .001 | 1.36 |  |
|  | ASRS Total | 0.20 | 0.08 | 0.31 | 2.68 |  | .010 | 1.37 |  |

*Note.* Full model refers to multiple linear regression including covariates (treatment assignment and treatment responder status) entered together with predictor variables. Final model refers to multiple linear regression with backward elimination – with covariates (treatment assignment, treatment responder status) entered into the model at each step and predictor variables with a p-value of < .05 removed from the model at each step in ordered fashion.

Treatment Assignment refers to randomization to Comprehensive Behavioral Intervention for Tics (coded as 1) versus Psychoeducation and Supportive Therapy (coded as 0). Treatment Responder Status refers to Clinical Global Impressions CGI-I rating of ‘much improved’ or ‘very much improved’ (coded as 1) relative to a rating of ‘improved,’ ‘minimally improved,’ ‘no change,’ ‘minimally worse,’ ‘much worse,’ or ‘very much worse’ (coded as 0). Sex is coded as 1 (male) and 0 (female). Medications are coded as 1 (on medication) and 0 (off medication).

YGTSS = Yale Global Tic Severity Scale; SCARED-Adult = An Adult Version of the Screen for Child Anxiety Related Disorders; MFQ = Mood and Feelings Questionnaire-Short; ASRS = Adult ADHD Self-Report Scale; PSQI = Pittsburgh Sleep Quality Index.

*β =* standardized beta (regression) coefficient, which ranges from -1 to 1; values nearer -1 or 1 indicate a stronger association between predictor and dependent variable. The alpha level was set to .05. VIF = variance inflation factor; lower scores indicate reduced multicollinearity among predictor variables. Adjusted R^2^ = predictive power of a model adjusted for the number of predictors in the model; higher values indicate greater predictive power based on the predictors in the model.
